# Being stressed outside the park—conservation of African elephants (*Loxodonta africana)* in Namibia

**DOI:** 10.1093/conphys/cox067

**Published:** 2017-12-18

**Authors:** Louis Hunninck, Iris H Ringstad, Craig R Jackson, Roel May, Frode Fossøy, Kenneth Uiseb, Werner Killian, Rupert Palme, Eivin Røskaft

**Affiliations:** 1 Norwegian University of Science and Technology—NTNU, Department of Biology, Høgskoleringen 1, 7491 Trondheim, Norway; 2 Norwegian Institute for Nature Research—NINA, Department of Terrestrial Ecology, Høgskoleringen 9, 7034 Trondheim, Norway; 3 Ministry of Environment and Tourism, Directorate of Scientific Services, P/Bag 13306, Windhoek, Namibia; 4 University of Veterinary Medicine, Veterinärplatz 1, 1210, Vienna, Austria

**Keywords:** conservation, human activity, stress, African elephant, faecal glucocorticoid metabolite, Etosha National Park

## Abstract

The conservation of the African savanna elephant (*Loxodonta africana*) is of prime importance for many African countries. Interactions between elephants and humans are known to induce stress and thereby have the potential to affect elephants’ fitness. In Namibia, anthropogenic disturbances are increasing due to increasing human population size and development, particularly near protected areas, such as national parks. In this study, we investigated elephant stress levels in relation to their land use, specifically their protection status, comparing elephants within Etosha National Park in Namibia with elephants residing outside the park. We noninvasively collected dung samples of 91 elephants and determined the concentration of faecal glucocorticoid metabolites (fGCM), an indicator of physiological stress. Elephants outside the park (*N* = 35) had significantly higher concentrations of fGCM than those inside ENP (*N* = 56), suggesting that, despite including community-based conservancies, unprotected areas are more stressful for elephants than protected areas, most likely due to increased interactions with humans. We also found that males had lower fGCM concentrations than females, but no significant effect of age, body size or group size was detected. Additionally, herd sizes were significantly smaller and calf recruitment was potentially lower in unprotected areas. These findings underpin the importance of protected areas such as ENP, while encouraging decision-makers to continue reducing and mitigating potential human-induced disturbances.

## Teaser

Human–wildlife interactions are of pressing concern for conservation biologists. We show that African elephants are chronically stressed, have smaller group sizes and may have lower calf recruitment outside of a protected area compared to inside. Additionally, this paper discusses some strengths and weaknesses of using faecal hormone analyses in the field.

## Introduction

In many parts of Africa, wildlife populations and the human communities living alongside them often compete over land and the natural resources therein. This often results in harmful interactions between them and can result in, among other, loss of crops or physical injuries (Madden, 2004; Sarker, 2010). These unwanted interactions only intensify with increasing human populations, and have become a pressing matter for many countries around the world. On the African continent, the African savanna elephant (*Loxodonta africana*) is facing many threats due to anthropogenic disturbances such as poaching and habitat degradation (Chase and Griffin, 2009; Wittemyer *et al.*, 2014; Thouless *et al.*, 2016). As an ecologically important species influencing ecosystem structure and function, as well as being a flagship species used to promote conservation efforts internationally (Barua, 2011; Haynes, 2012), their preservation and sustainable management is of major importance, especially in those countries where elephant populations are plummeting (Chase *et al.*, 2016). Mitigating human-induced disturbances (Young *et al.*, 2010) is not only beneficial for elephant populations, but for the local communities living alongside them, too (Sarker, 2010; Hariohay and Røskaft, 2015). Additionally, harmful interactions cause negative human attitudes towards elephants, which aggravates the problem (Newmark *et al.*, 1993; Sitati *et al.*, 2003; Inogwabini *et al.*, 2013). The competition with human populations affects wildlife populations in various ways; in this study, we quantified stress hormones in elephants’ faeces as indicator of their physiological stress in order to measure the potential impact of the anthropogenic disturbances on elephant populations.

When exposed to unpredictable or high-risk situations called ‘stressors’, an animal will react with a stress response (Romero, 2004). This stress response can have a range of effects, including behavioural and physiological changes (Romero and Wingfield, 2016). Failing to mitigate the stressor (e.g. by dispersing) will result in a continuous and long-term activation of the endocrine and metabolic stress response (i.e. chronic stress) which can cause a reduction in an animal’s fitness (Pride, 2005; Busch and Hayward, 2009; Mumby *et al.*, 2015). Romero (2004) provides a good introduction and review of the complexity of the physiological stress response. The physiological stress response is different with respect to different intensities of stressors. In general, more severe stressors induce a greater release of glucocorticoids (GC) in the bloodstream through increased stimulation of the hypothalamic-pituitary-adrenal axis, which in turn results in a greater concentration of metabolized GC in faeces (Romero, 2004). When the stressor ceases, GC concentrations normally return back to pre-stressor levels. This process is an evolutionary adaptation to successfully handle stressful situations. Stress can be measured by observing behavioural alterations, though in some contexts, this can be subject to misinterpretation (Caro, 1999; Rushen, 2000). Measuring physiological changes is another method to observe stress, and can be a valuable technique in addition to observing, for example, anti-predator and movement behaviour (Möstl and Palme, 2002). Measuring the concentration of faecal glucocorticoid metabolites (fGCM) is a widely used and non-invasive method to quantify stress levels (Millspaugh *et al.*, 2001; Creel *et al.*, 2002; Viljoen *et al.*, 2008; Sheriff *et al.*, 2009, 2011; Tingvold *et al.*, 2013). Additionally, it allows for a more accurate representation of stress over a longer period of time (i.e. chronic stress) compared to invasive techniques such as blood sampling (Palme *et al.*, 2005). This is due to the time it takes for faeces to pass through the digestive system (i.e. gut transit time) (Palme, 2005), which in elephants takes about 2–3 days (Wasser *et al.*, 2000). The measurement thus reflects the average concentrations of glucocorticoid stress hormones circulating in the body during gut transit time and is less likely to detect acute increases in stress levels but will rather detect a stressor of longer duration, such as a steady elevation of stress hormone concentrations due to a continuous disturbance (Goymann, 2005; Harper and Austad, 2012).

Apart from natural stressors such as drought and predation, stressors can include various anthropogenic disturbances. These can be due to the indirect effects of, among other, habitat destruction or roads, or due to direct interactions with humans, such as those experienced by working elephants (Millspaugh *et al.*, 2007) and crop-raiding elephants (Ahlering *et al.*, 2011). Adjacent to national parks and other protected areas, human populations are growing due to increased prosperity from tourism (Andrade and Rhodes, 2012), and often experience increased rate of human–wildlife interactions as a consequence of wildlife emigrating from the protected areas (Vedeld *et al.*, 2012). Protected areas aim to preserve and protect biodiversity and ecosystem services mainly by minimizing negative anthropogenic impacts on wildlife. Consequently, wildlife inhabiting protected areas with tourism as main land use may have lower stress levels than conspecifics residing outside the protected area, where the dominant land use is agro-pastoralism and human habitation (Romero and Wingfield, 2016). Differences in elephant stress levels inside and outside protected areas may vary greatly between ecosystems, and would depend on the type and extent of human activities. For example, there was no evidence of chronic stress in elephants occurring in community conservation areas (CCA) outside Amboseli National Park, Kenya (Ahlering *et al.*, 2013). These CCA are partially protected areas that act as buffer for neighbouring national parks and, among others, aim to prevent loss of biodiversity and mitigate negative human–wildlife impacts. The researchers thus concluded that their results were encouraging for current conservation efforts. Elephants immediately outside of Serengeti National Park, Tanzania, on the other hand, were significantly more stressed than elephants inside the park (Tingvold *et al.*, 2013). Although those areas were also partially protected as Game Reserves, this did not seem to prevent an increased stress response.

In many ungulates, anthropogenic disturbances have been thought to be perceived in a similar manner as predator cues, and anti-predator behaviours could consequently be observed in ungulates attempting to minimize the disturbance (Frid and Dill, 2002). Such behaviours include vigilance rates and the distance at which animals flee from a potential threat. However, although some ungulate species form larger groups when subjected to higher predation pressure (Hunter and Skinner, 1998), the opposite effect is often observed when subjected to anthropogenic disturbance (Setsaas *et al.*, 2007; Averbeck *et al.*, 2010; Kioko *et al.*, 2013). This might simply be due to increased illegal killing of wildlife in unprotected areas, or it might be a behavioural adaptation to increased anthropogenic disturbance. Regardless, group size and structure are important indices to consider for conservation management as they can affect population growth rates and extinction risks. African elephants have been observed to increase their reproductive effort in relation to increased mortality (Wittemyer *et al.*, 2013), and when severe pressures such as intensive poaching are removed, elephants have the potential to increase rapidly in numbers (Foley and Faust, 2010). In this study, we observed elephant group size and calf recruitment to assess if these variables were affected by increased levels of anthropogenic disturbance. Calf recruitment was measured by estimating the number of calves per adult female in a group (see ‘Methods’).

In Namibia, crop raiding and competition over water resources are the main contributors to harmful human–elephant interactions (Cumming and Jones, 2005; Jones and Barnes, 2006). We refrain from using the term ‘human–elephant conflict’ as this could imply that elephants are ‘conscious human antagonists’ (Peterson *et al.*, 2010), which can contribute to negative sentiments towards them and thus obstruct peaceful human–wildlife coexistence (Redpath *et al.*, 2015). We do recognize human–human conflicts in elephant conservation (Young *et al.*, 2010), such as those between conservationists and poachers. Although poaching poses a considerable threat to wildlife in general (Gavin *et al.*, 2010; Wittemyer *et al.*, 2014; Chase *et al.*, 2016), it probably does not have a major impact on elephants in this area anymore, since poaching is considered minimal (CITES, 2016a). However, especially outside of Etosha National Park (ENP), elephant populations have been under extreme pressures from hunting and military conflict up until 1990, removing almost all elephants west from ENP, the Kunene region (KR) (Lindeque, 1988; Martin, 2005). Current population structure and densities of elephants residing in the KR are thus likely to still be affected by this historical disturbance.

Cooperation and willingness of the local communities to protect the elephant are essential if conservation objectives set by wildlife managers are to be met (Berkes, 2004). To that end, currently 82 areas, which include human settlements, have been declared conservancies in Namibia, to partially protect wildlife in areas outside of national parks (Weaver and Skyer, 2003; MET Conservancies, 2017). These areas cover about 19.8% of Namibia’s total area, and this is in addition to the 16.8% designated as protected area (MET Conservancies, 2017). The conservancies have the primary goal to help mitigate the conflict between wildlife and human communities, and to involve local communities in conservation and the profits thereof. In return, the conservancies need to, among other, have a wildlife management plan, need to aid MET with annual surveys, and regulate wildlife exploitation (Tambara *et al.*, 2016). According to CITES, the current elephant trophy hunting quota in Namibia is 180 tusks (or 90 individuals) annually since 2005 (CITES, 2017).

Here, we investigated fGCM concentrations in elephants located in conservancies in the Kunene region, north-west Namibia, and in the adjacent ENP. We hypothesized that, even with the partially protected status of the conservancies, mean stress levels would be higher outside the strictly protected ENP than inside due to higher anthropogenic disturbances outside. Additionally, we expected that group sizes would be lower, and that their calf recruitment would be higher, due to higher past and present legal and illegal killing of individuals outside of the park. This research contributes to a better understanding of the physiological stress elephants experience in areas with anthropogenic disturbance. It can be used to gauge the current efficacy of wildlife management in north-western Namibia and to assess whether conservation goals are met.

## Materials and methods

### Study area and anthropogenic disturbance

**Figure 1:**
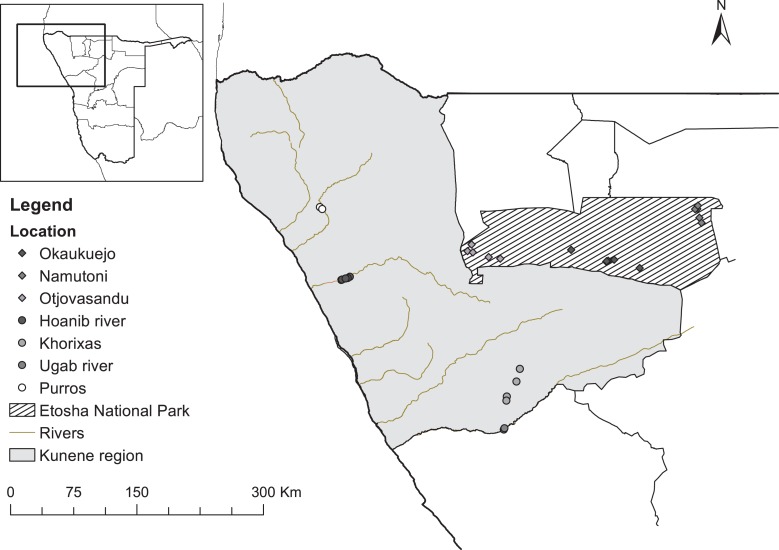
Map showing the sample locations inside Etosha National Park and in the Kunene region, located in north-west Namibia. In the Kunene region, samples were often taken close to rivers. *Data source: Environmental Information Service (EIS), Namibia, 2016*http://www.the-eis.com.

To distinguish between different levels of anthropogenic disturbance, we selected two contrasting areas inhabited by elephants that differed significantly in several proxies of anthropogenic disturbance: Etosha National Park and the Kunene region. Three protected locations, spanning a geographical east-west gradient, were sampled inside ENP, in addition to four locations outside the ENP boundary in the KR; the latter were selected based on information from rangers on elephant distribution (Fig. 1). The completely fenced ENP is one of Namibia’s largest national parks (18 549 km^2^) (Boyle *et al.*, 2015; Thouless *et al.*, 2016). It has an arid climate (mean annual rainfall is 430 mm) and an extensive saltpan (2800 km^2^). The park is also inhabited by 2911, SE = 637 resident elephants (as of 2015) (Thouless *et al.*, 2016). CITES’s Monitoring of Illegal Killing of Elephants (MIKE) program has not recorded any illegally hunted elephants within ENP between 2002 and 2015 (Malpas and D´Udine, 2013; CITES, 2016b). We considered ENP as a safe habitat for elephants with minimal levels of anthropogenic disturbance, because of its protected status and strict regulations. In contrast, the KR, bordering ENP to the south and west, has high human presence, abundant livestock rearing and presence of human-made waterholes constructed for livestock, in addition to sustainable hunting practices, poaching, and less controlled, high-impact tourism. Although the KR consists mostly of designated conservancies, we expected that elephants, although lower in numbers and density (314, SE = 154; as of 2011) in the KR (Thouless *et al.*, 2016), experience more negative interactions with humans and therefore have higher concentrations of glucocorticoid stress hormones compared to those residing within ENP.

### Data collection

Data was collected by visiting known elephant ranges between June and August 2014. We encountered 392 elephants in areas inside and outside ENP, and, by visiting a sampled group not more than once and photographing every individual, collected faecal samples from 91 unique individuals. Data inside and outside the park was collected in one session and each within 16 and 21 days, respectively. Most samples were collected near waterholes or riverbeds where it was easier to find and observe elephant groups.

For each sample, group size (3 categories: single male, 2–15, and 16+ individuals), body size, sex (for 7 juveniles, sex could not be determined), age, date and time, GPS coordinates, and ambient temperature were recorded. A distinction was made between bull groups (only male individuals) and family herds (possibly including both sexes but predominantly female). The sex of an individual was determined according to Moss (1996); the age of an individual (adult, sub-adult, juvenile or calf) was based on body size, tusk length, head and back shape (Moss, 1996). Where several herds mixed together, group size was recorded as the total number of individuals. Body size was measured as a relative percentage to the biggest female (often matriarch) defined as 100% and the remainder of individuals assessed accordingly, adult males typically at 200% and juveniles at 40% (Tingvold *et al.*, 2013). The hierarchical position of individuals (e.g. matriarch) was not determined, and only very few males that were sampled showed signs of musth (determined by observing secretions of the temporal glands; Poole, 1987), though this has been shown not to increase physiological stress levels (Ganswindt *et al.*, 2010).

### Faecal sample protocol

Our sampling protocol followed Tingvold *et al.* (2013). Fresh dung samples were collected from observed individuals with dung never being exposed more than 2 h to minimize environmental degradation of the glucocorticoid metabolites in the samples. We ensured that no samples subjected to rain or urine were collected. To minimize confounding effects and misinterpretation of the data, we recorded time between defecation and collection (here referred to as delay-time), ambient temperature and time of day at collection, and tested whether these significantly affected fGCM concentrations (*see also* Baker *et al.*, 2013). For each defecation, the outer layer (including mucus deposit) of 3–4 boli was sampled (around 30 ml) to account for potential variation in metabolite distribution between boli in the faeces. This way, a potential acute stressor is less likely to be observed in the sample. After collection, the sample was immediately frozen in a portable freezer at −18°C before moved to a permanent freezer at −20°C.

### Lab procedures

The lab procedures for initial extraction of the glucocorticoid metabolites was done according to Palme (2005) and Touma and Palme (2005). After defrosting at room temperature (up to 30 min), the faecal samples were homogenized thoroughly by hand for 5 min and, excluding undigested materials, a 0.5 gr (±0.02) subsample was put into a 15 ml centrifuge tube. About 5 ml of 80% methanol was added and the tubes were subsequently vortexed for 3 min. After centrifuging for 20 min at 1500 rpm, 0.5 ml supernatant was extracted. Vials were left opened under a fume hood to dry out (up to 2 days). Samples were sealed and stored at room temperature until further analysis. GC metabolites were measured with an 11-oxoaetiocholanolone EIA (first described by Möstl et al., 2002) which measures metabolites with a 3α-hydroxy-11-oxo structure. This EIA has been successfully validated for African elephants (Ganswindt et al., 2003).

### Statistical analyses

The fGCM concentrations were log-transformed to obtain normal distribution, as is standard procedure with hormone data (Creel *et al.*, 2002; Munshi-South *et al.*, 2008; Tingvold *et al.*, 2013; Munerato *et al.*, 2015). Linear multivariate mixed regression models (*lmer* function, ‘lme4’ package) were developed using the package MASS (Venables and Ripley, 2002). The different models were *a priori* selected based on biological relevance, with fGCM level as the response variable, and land use (i.e. ENP or KR), sex, group size, body size, age, delay-time, time of day, ambient temperature as fixed predictor variables. Within most sampling locations, faecal samples were collected from multiple groups. Therefore, to control for between-group and between-location variation, we included group-ID nested within sampling location as random effect. The resulting models were compared using AICc (Akaike’s Information Criterion adjusted for small samples sizes) to determine the most parsimonious model that explained most of the variation in the data (Table 1) (Johnson and Omland, 2004).

**Table 1:** Best models based on AICc selection with respective ∆AICc value. Detail of the two most parsimonious linear mixed models including five variables affecting fGCM concentrations (KR = Kunene region). fGCM concentration is log-transformed

Model structure					∆AICc
log(fGCM) ~ Land use + Sex + Delay + (1|Location/Group)					0.217
log(fGCM) ~ Land use + Delay + (1|Location/Group)					0.023
**Fixed**	Estimate	Std error	*t*-value	*P*-value	
*(intercept)*	3.938	0.109	36.30	<0.001	
Land use—KR	0.395	0.129	3.058	0.007	
Delay	0.004	0.002	1.946	0.067	
**Random**	Variance	Std Dev			
Group:Location	0.035	0.187			
Location	0.000	0.000			

log(fGCM) ~ Land use + Sex + (1|Location/Group)					0.000
**Fixed**	Estimate	Std error	*t*-value	*P*-value	
*(intercept)*	3.938	0.109	36.30	<0.001	
Land use	0.356	0.141	2.53	0.018	
Sex—Male	−0.222	0.112	−1.989	0.050	
**Random**	Variance	Std Dev			
Group:Location	0.064	0.252			
Location	0.000	0.000			

Data for group size consisted of count data, which is better modelled by a Poisson or negative binomial distribution (Bolker *et al.*, 2009), and for this particular model, we used the latter distribution. Consequently, we used a generalized linear mixed model (*glmer.nb* function, ‘MASS’ package), with family herd group size as response variable and land use as predictor variable; sample location was modelled as a random effect.

The measure for female reproductive success was derived by calculating the number of calves per adult female in a herd, which resulted in a strictly positive and continuous variable. To obtain normally distributed data, we log-transformed this response variable (i.e. calves per adult female) and used a linear mixed regression model (*lmer* function, ‘lme4’ package) with land use as predictor variable and sampling location as random effect.

Residuals of the statistical models used in the analyses were all normally distributed. All statistical analyses were performed in RStudio (R Version 3.3.1 GUI 1.68) (RStudioTeam, 2015).

## Results

**Figure 2:**
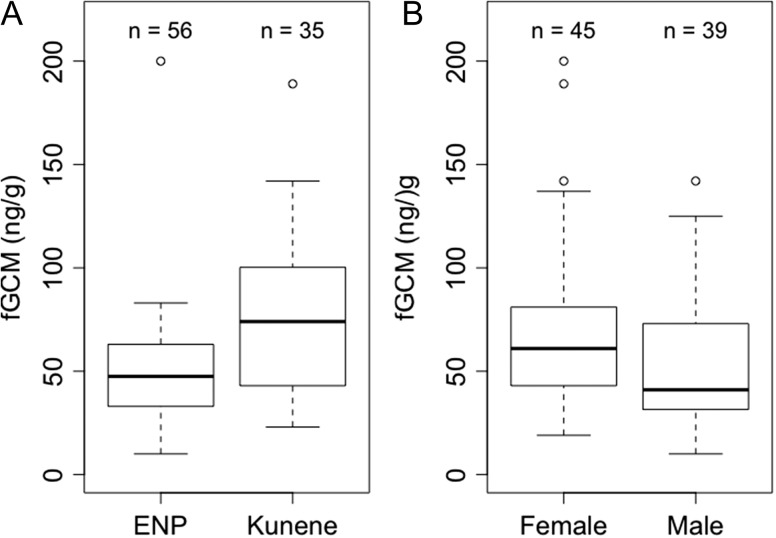
fGCM concentrations for (**A**) African elephants in the strictly protected ENP and the Kunene region, and (**B**) female and male elephants (lower and upper lines of boxes, 25th and 75th percentiles, respectively; solid line, median; whiskers, maximum and minimum values; circles, potential outliers).

**Figure 3:**
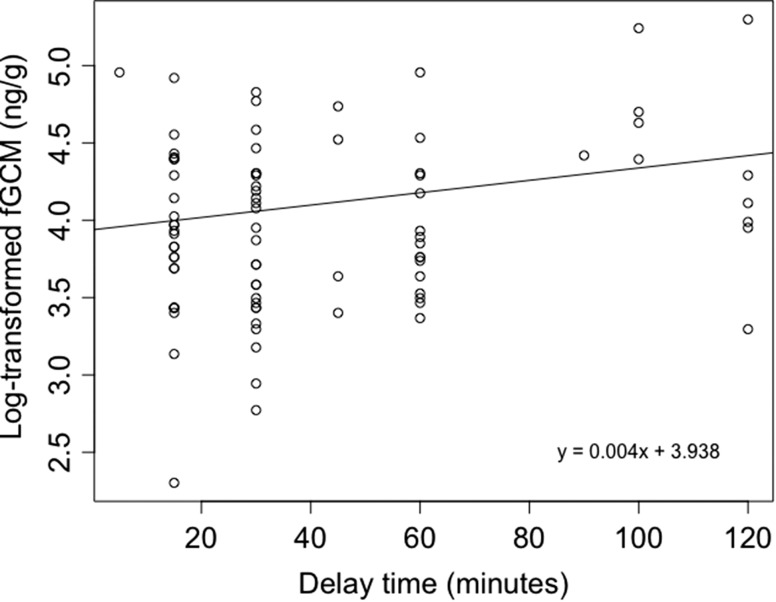
Regression analysis showing the effect of delay-time (i.e. time between defecation and storage in freezer) on the fGCM concentrations in elephants.

Mean concentrations of fGCM were significantly higher (51%) in elephants sampled outside ENP than those sampled inside the protected area (Table 1, Fig. 2). Additionally, female elephants showed significantly higher (27%) stress levels than males (Table 1, Fig. 2). Conversely, we found no association between fGCM concentrations and either age, body size, or group size. The models with land use and sex, land use and delay-time, and land use, sex and delay-time, could not be statistically distinguished from each other (i.e. ∆AICc < 2). With increasing delay-time, there was a small, marginally significant increase in measured fGCM concentrations (Table 1); delay-time ranged from 5 to 120 min (mean = 42.36, SD = 30.83; Fig. 3). Ambient temperature at the time of collection, and diurnal patterns in sample collection did not significantly affect fGCM concentrations.

During the 32 field days, we observed 392 elephants in 45 groups and collected 91 faecal samples from these. Faecal glucocorticoid concentrations ranged from 10 ng/g to 200 ng/g throughout the whole sample pool, with a mean of 60.91 ng/g, SD = 35.07 (*N* = 91). Family herd sizes (predominantly female) were significantly smaller outside ENP than inside (*χ*^2^ = 7.55, df = 1, *P* = 0.006, *N* = 26), with 15 individuals being the largest group encountered outside ENP (median = 8.5, min = 2), compared to 36 individuals inside ENP (median = 13.0, min = 6). The number of calves per adult female tended to be lower outside ENP (mean = 0.26, SE = 0.07) compared to inside (mean = 0.46, SE = 0.02; *t*-value = −1.993, *P* = 0.064).

## Discussion

Anthropogenic disturbances can be perceived as a stressful experience for elephants and as such can induce a physiological stress response, thereby increasing the concentration of stress hormones in the body. Though we did not measure anthropogenic disturbance directly, since both areas differ significantly in various disturbance-related factors such as human habitation, hunting pressure, and land use, we concluded that a qualitative distinction sufficed to examine the potential impact of anthropogenic disturbances. We hypothesized that elephant populations in the partially protected KR had increased levels of stress caused by anthropogenic disturbances including human–elephant impacts (crop raiding and physical injuries) compared to elephants inside ENP (Millspaugh and Washburn, 2004). Our results partly support this hypothesis, as elephants residing within the partially protected KR had higher fGCM concentrations than elephants living inside ENP (Table 1, Fig. 2). Our results therefore corroborate other studies which have found comparable effects of protection status on elephant stress levels (Millspaugh *et al.*, 2007; Gobush *et al.*, 2008; Jachowski *et al.*, 2013b; Tingvold *et al.*, 2013), although the type of human-related stressor has differed. However, we could not fully control for other potential factors influencing the observed elevated stress response, apart from anthropogenic disturbances, such as the availability of water. It is important to note that, although the MIKE program did not record any poached elephants during and before this study was conducted, since then, illegal killing of both black rhinoceros (*Diceros bicornis*; Muntifering *et al.*, 2017) and savanna elephants has increased in Namibia, potentially aggravating stress experienced by elephants residing in those areas with increased poaching.

Elephants are heavily dependent on the availability of surface water, and since this study was conducted during the early dry season, the low amount of surface water could have contributed to elevated stress levels as a natural stressor (Foley *et al.*, 2001; Touma and Palme, 2005; Viljoen *et al.*, 2008). While there are multiple waterholes providing nearly year-round availability of water designated for wildlife in ENP, there are few natural water sources in the KR, most of which only provide water in the rainy season. Most waterholes and other water sources during the dry season are therefore human-made and designed for pastoralist livestock and human use. Although elephants have developed ways to minimize direct contact with humans by, for example, visiting water points after sunset when human activities are reduced, or avoiding roads during daytime, these water sources are regularly utilized by elephants, which increases their confrontation rate with humans. The preferential use of communal lands by elephants has been shown by Graham *et al.* (2009), who found that the more communal land was available to elephants, the more they utilized it, potentially increasing negative interactions with humans. Thus, the search for water, which might increase stress levels in and of itself, is often closely associated with human interactions and could, consequently, result in additional human-induced stress.

Stress levels differed significantly between males and females, with males having lower mean stress levels than females (Table 1, Fig. 2). This might be due to physiological differences between the sexes (Creel *et al.*, 2013) where females have for example the additional burden of raising calves and/or are responsible for the herd. Differences in reproductive state of individuals could also affect this, though the females sampled for this study did not show signs of pregnancy or lactating (Rasmussen *et al.*, 2008). Touma and Palme (2005) review several physiological differences between males and females that could explain the observed difference. These include differences in steroid-binding proteins with high affinities for GCs, and a difference in proportion of GCs eliminated in faeces and urine. Other researchers found no significant difference between sexes (Munshi-South *et al.*, 2008; Tingvold *et al.*, 2013), while others found the opposite effect (Ahlering *et al.*, 2013). The relation between sex and stress response is highly species dependent and in elephants, still unclear.

The relationship between observed stress levels and perceived anthropogenic disturbance may not always be intuitive. Munshi-South *et al.* (2008) found that forest elephants (*L. cyclotis*) inside a protected area had higher fGCM concentrations than elephants outside of the protected area. Ahlering *et al.* (2013) found that elephants residing in a community conservation area (CCA) did not show elevated stress levels compared to one (of two sampled) protected area(s), although this could indicate that the conservation efforts in the CCA are sufficient with respect to reducing chronic stress in elephants residing in those areas. In addition, according to Grissom and Bhatnagar (2010), elephants may have become habituated to the presence of humans and their vehicles when the disturbance is not deemed noxious, which could have happened in the protected ENP where tourism is the only major activity, thereby lowering fGCM concentrations. To avoid misinterpreting stress hormone data, study design and protocols should consider the factors influencing the concentration of stress hormones in the faeces, including the methods of collecting, storing, and analysing the samples (Palme, 2005; Baker *et al.*, 2013; Wilkening *et al.*, 2016; Ranglack *et al.*, 2017). The samples in Ahlering *et al.* (2013) were collected up to 12 h after defecation and environmental degradation of samples due to bacterial activity can be substantial (Palme, 2005). The effect of this degradation is unpredictable, depending on the metabolites excreted and the method used to analyse the samples (i.e. type of anti-body), the direction of change can be both positive and negative (Romero and Wingfield, 2016). Despite that our samples were collected within 2 h after defecation, we found tentative evidence of a temporal positive degradation effect. With increasing time between defecation and storage of the sample on ice, fGCM concentrations marginally increased (Fig. 3). This environmental degradation is an important yet underestimated drawback of analysing faecal samples for hormone, and although this tool has some excellent advantages (not in the least its non-invasive nature), its weaknesses need to be carefully considered when preparing study design and sampling protocol.

Elevated stress levels can affect an individual’s physiological functions (Romero, 2004; Mumby *et al.*, 2015) and increased long-term stress levels could lead to a fitness reduction (Pride, 2005; Jachowski *et al.*, 2013a). This could potentially affect an animal’s fertility and reproductive success resulting in a decreased population size and persistence. We found that elephant herd size was significantly smaller outside the protected area, which could suggest, among other, a lower maximum carrying capacity of the area, or a higher calf mortality rate. Whether the decreased group size is due to increased levels of chronic stress cannot be assessed by the data collected for this study, but should be further investigated. Similarly, the potential effect of smaller group sizes on stress levels could not be determined in this study, though group size has been found to correlate negatively with fGCM concentrations (Foley *et al.*, 2001). African elephants were reported to have a greater fecundity (measured by primiparous age and inter-calf interval) in human disturbed areas compared to stable populations in Kenya (Wittemyer *et al.*, 2013). Here, we found that family groups outside the protected areas tended to have, on average, almost half as many calves per adult female. Stress-related causes for this comparatively low number of calves per adult female cannot be excluded, since elevated stress levels have been shown to inhibit behavioural and physiological aspects of reproduction (Sapolsky *et al.*, 2000; Creel *et al.*, 2013; Romero and Wingfield, 2016), but cannot be assessed with the data presented here. Further research on the ultimate effects of elevated stress levels on individual fitness and population viability would allow this already frequently used sampling technique to become even more helpful in conservation biology. In their review study, Busch and Hayward (2009) found ambiguous relations between fGCM concentrations and fitness, and the threshold at which elevated fGCM concentrations become harmful are not yet fully elucidated (Millspaugh and Washburn, 2004). Furthermore, apart from a good understanding of the complex physiological mechanisms and potential confounding effects, to effectively review studies concerning stress hormones, standardized and comparable methodology is essential since small differences in collection and analysis protocol can yield very different recovery rates of hormone metabolites and comparisons of absolute stress hormone metabolite concentrations in different studies can therefore be misleading (Keay *et al.*, 2006).

Further studies conducted in this system should span a longer time period, including the wet season, to control for stress induced by decreased water and food availability. Additionally, since thyroid hormones have been shown to correlate with nutritional stress but not with physiological stress (Wasser *et al.*, 2010), including the analysis of thyroid hormones in the faecal samples would increase the robustness of the findings. Nonetheless, our results suggest that elephants experience elevated chronic stress levels in areas where anthropogenic disturbance is significant. It seems that, even in a country with low human population density, minimal poaching and community-based conservation schemes, elephant populations can still be affected by increased chronic stress. Due to the many potential negative consequences related to chronic stress, such as reducing overall fitness and affecting population viability, efforts to improve an animal’s potential stressors is vital when considering conservation measures. This research acknowledges the importance of protected areas such as ENP, and stresses that further mitigation of the anthropogenic disturbances are pivotal for the long-term survival of elephant populations that will inevitably reside outside those protected area boundaries.

## Acknowledgments

The study was done upon request and in collaboration with the Ministry of Environment and Tourism (MET) in Namibia, which issued the necessary research permits. Logistical support during fieldwork was offered by MET staff, and in particular Chief Warden Shane Kötting and Warden Oiva Akudhenga. Export permits to transport biological samples out of Namibia were issued by Convention on International Trade in Endangered Species of Wild Fauna and Flora (CITES).
