# Being stressed outside the park—conservation of African elephants (*Loxodonta africana)* in Namibia

**DOI:** 10.1093/conphys/cox080

**Published:** 2018-01-25

**Authors:** Louis Hunninck, Iris H Ringstad, Craig R Jackson, Roel May, Frode Fossøy, Kenneth Uiseb, Werner Killian, Rupert Palme, Eivin Røskaft

*Conserv Physiol* (2017) 5(1): cox067; doi: 10.1093/conphys/cox067

The original article omitted the name of a co-author, Professor Rupert Palme, and two references. The article has been updated to include this information, and an update to the Material and Methods section. For the purposes of transparency, the changes were as follows:

The text reading ‘GC metabolites (3α,11-oxoaetiocholanolone cortisol metabolites) were extracted using an enzyme immunoassay (EIA; 5β-Androstane-3α-ol-11-one-17-CMO, developed by E. Möstl and R. Palme from the ICN 07-120 102 corticosterone antibody [Wasser *et al.*, 2000]), a method specially developed for GC metabolites and validated (reliably detecting adrenal activity in faeces) for African elephant (Wasser *et al.*, 2000).’ has been replaced with ‘GC metabolites were measured with an 11-oxoaetiocholanolone EIA (first described by Möstl *et al.*, 2002) which measures metabolites with a 3α-hydroxy-11-oxo structure. This EIA has been successfully validated for African elephants (Ganswindt *et al.*, 2003).’

The following references were also inserted at the end of the paper:

‘Möstl E, Maggs JL, Schrötter G, Besenfelder U, Palme R (2002) Measurement of cortisol metabolites in faeces of ruminants. *Vet Res Commun* 26:127–139.’

‘Ganswindt A, Palme R, Heistermann M, Borragan S, Hodges JK (2003) Non-invasive assessment of adrenocortical function in the male African elephant (Loxodonta africana) and its relation to musth. *Gen Comp Endocrinol* 134:156–166.’

The authors would like to apologise for these errors.

